# Potential Effects of a Modified Mediterranean Diet on Body Composition in Lipoedema

**DOI:** 10.3390/nu13020358

**Published:** 2021-01-25

**Authors:** Laura Di Renzo, Giulia Cinelli, Lorenzo Romano, Samanta Zomparelli, Gemma Lou De Santis, Petronilla Nocerino, Giulia Bigioni, Lorenzo Arsini, Giuseppe Cenname, Alberto Pujia, Gaetano Chiricolo, Antonino De Lorenzo

**Affiliations:** 1 Section of Clinical Nutrition and Nutrigenomic, Department of Biomedicine and Prevention, University of Rome Tor Vergata, Via Montpellier 1, 00133 Rome, Italy; nsanfelice02@gmail.com (P.N.); delorenzo@uniroma2.it (A.D.L.); 2 School of Specialization in Food Science, University of Rome Tor Vergata, 00133 Rome, Italy; giuliacinelli88@gmail.com (G.C.); lorenzo.romano@alumni.uniroma2.eu (L.R.); samanta.zomparelli@gmail.com (S.Z.); gemmaloudesantis@gmail.com (G.L.D.S.); 3 Predictive and Preventive Medicine Research Unit, Bambino Gesù Children’s Hospital IRCCS, 00165 Rome, Italy; 4 Department of Physics, University of Rome Sapienza, 00185 Rome, Italy; bigionigiulia@gmail.com (G.B.); lorenzo@arsini.it (L.A.); 5 General Command of the Carabinieri, Health Department, 00197 Rome, Italy; giuseppe.cenname@carabinieri.it; 6 Department of Biomedicine and Prevention, University of Rome Tor Vergata, 00133 Rome, Italy; albpujia@gmail.com (A.P.); nucciochiricolo@gmail.com (G.C.)

**Keywords:** Lipoedema, SAT diseases, antioxidant diet, body composition, nutrition

## Abstract

Lipoedema is a subcutaneous adipose tissue disease characterized by the increase in the amount and structure of fat mass (FM) in specific areas, causing pain and discomfort. 95% of patients fail to lose weight in the lipoedema areas. The study was conducted to evaluate body composition and general health status modification in a group of lipoedema patients (LIPPY) and a control group (CTRL) after four weeks of a modified Mediterranean diet therapy (mMeD). A total of 29 subjects were included in the data analysis, divided in two groups: 14 LIPPY and 15 CTRL. After the mMeD, both groups significantly decreased their weight and body mass index; the CTRL also showed a reduction of all the circumferences and all FM’s compartments. LIPPY showed a decrease of FM in upper and lower limbs. No significant differences in Δ% between the groups were observed for the lean mass (LM). In LIPPY, an increase in the patients’ ability to perform various daily physical activities related to the loss of arms’ and legs’ fat was observed. According to the European Quality of Life scale, the possibility for LIPPY subjects to perform simple daily activities with less fatigue, pain and anxiety is highlighted. Further long-term studies are recommended to confirm the mMeD as a good strategy for Lipoedema treatment.

## 1. Introduction

Subcutaneous Adipose Tissue (SAT) diseases are characterized by the increase in the amount and structure of fat, causing pain and discomfort. In SAT diseases, fat is resistant to weight loss following lifestyle interventions, dietary changes, surgery or pharmacologic therapy due in part to tissue fibrosis. For this reasons, SAT in SAT diseases is considered a persistent fat [1].

Although lipoedema is mostly an underrecognized condition often misdiagnosed as lymphedema or simple obesity, Child et al. presented lipoedema as a genetic condition with X-linked dominant inheritance or, more likely, autosomal dominant inheritance with sex limitation [2] to mostly women. The same authors revealed a prevalence within the population as 1:72,000 and men with lipoedema are extremely rare [3]. In lipoedema, lymph vessels are unable to maintain their function. The altered microcirculation leads to impaired lymph transport capacity and accumulation of lymph fluid. The high protein and fat content of lymph fluid evokes subsequent fibrosis and further deposition of fat leading to non-pitting edema characterized by the Stemmer’s sign [4].

Lipoedema is classified in stages according to Herbst et al. [5], considering the quality of the skin, the presence of fibrosis, the development of a nodular or mass-like appearance of subcutaneous fat, lipomas and/or angiolipomas, inhibition of mobility and coexistence of lymphedema. Most of the patients report onset of lipoedema at menarche, with progression of the disease dependent on family history and lifestyle. Lipoedema is a disease characterized by an inflammatory condition [6,7]. From a pathophysiological point of view, the progression of lipoedema is caused by dilation and lengthening of the lymphatic channels with the development of microaneurysms. The breakdown of microaneurysms combined with the increase in interstitial fluid leads to the development of lipo-lymphedema (the last stage of lipoedema) [8]. It has been suggested that regulation of steroid hormone levels by aldo-keto reductase 1C1 (AKR1C1) plays an important role in the accumulation of subcutaneous adipose tissue. The results are consistent with AKR1C1 being the first candidate gene for lipoedema [9]. Moreover, it has been observed that the IL-6 gene polymorphism characterizes subjects with lipoedema in comparison with normal weight obese people and/or obese subjects [10,11].

Even if lipoedema is a SAT disease, body composition study is extremely useful to provide information about fat mass (FM), lean mass (LM) and bone mass of the body and subregions. A case–control study of Dietzel et al. [12] showed the usage of dual-energy X-ray absorptiometry (DXA) not only for the diagnosis of lipoedema, but particularly for monitoring specific interventions including physical exercise. Thus, changes in the amount of FM and/or LM can be quantified as objective measurable parameters at an early stage. Considering the connection between lymphatic dysfunction, adipocyte hypertrophy of SAT and the progression to lipo-lymphedema, the interventions usually applied to treat lipoedema aim to support the lymphatic flow [8]. Although lipoedema fat is resistant to lifestyle changes, there is evidence to support positive effects of exercise, particularly of aquatic therapy [13]. Due to altered microcirculation, pain, lack of mobility and psychological reasons, lipoedema management aims to facilitate patients’ self-care ability, optimize health, prevent progression and modulate symptoms [14]. Psychological support and self-care, weight management, skincare protection and compression therapy are the main sectors of intervention to manage patients with lipoedema.

Improved understanding of dietary impact on outcomes of lipoedema may increase scientific and clinical awareness about the importance of nutritional approaches as well as provide directions for future research and strategies to prevent progression of this disease and the related pathologies.

Up to now, no effective nutritional treatment has been reported in patients with lipoedema, as no controlled trials have been published on this topic. Current dietary approaches are generally based on empirical data and are aimed at lowering body weight through a hypocaloric diet, inhibiting systemic inflammation with antioxidant and anti-inflammatory components and reducing water retention [15]. Literature data show that weight loss did not demonstrate any significant effect on the lipoedema prognosis due to fat deposition [16]. Lipoedema fat is resistant to diet therapy and 95% of patients fail to lose weight in the lipoedema areas [17]. In insulin-resistant subjects, enhanced lipolysis and impaired lipogenesis in adipose tissue lead to the release of cytokines and lipid metabolites, ultimately promoting insulin resistance. Therefore, since no specific diet has been developed for lipoedema so far, an isoglycemic diet would seem appropriate [18]. Accordingly, the few approaches studied in literature mainly involved diets with low content of processed carbohydrates with the effect of reducing inflammation and insulin levels and therefore adipogenesis [1]. For example, it has been hypothesized that the ketogenic diet may be efficient in the treatment of lipoedema in terms of weight loss, reduction of edema and modulation of the inflammasome with consequent improvement of the redox state [19]. The Mediterranean diet (MD) represents a dietary pattern associated with health benefits [20], with efficacy for patients with obesity and metabolic syndrome [21]. It has been reported that micronutrients of the MD modulate the immune system and exert a protective action reducing postprandial oxidative stress and inflammation [20]. In fact, micronutrients such as polyphenols, tocopherols, resveratrol, vitamin C, vitamin A increase the antioxidant capacity of the meal and the subject’s plasma antioxidant capacity [22]. Furthermore, they are able to modulate the expression of inflammation and oxidative stress-related genes [23].

Therefore, we hypothesized that a modified Mediterranean diet therapy (mMeD) based on the typical foods of the MD, such as fruit, vegetables, legumes, whole grains, extra virgin olive oil, fish and low-fat dairy products, could be adapted for patients with lipoedema.

The main purpose of the current study was to evaluate the effects of a mMeD on weight and body composition in women with lipoedema (LIPPY—the name was chosen by the Lipoedema Italia Onlus (LIO) association for women with lipoedema) compared to a control group (CTRL). In particular, the study focuses on the FM loss in upper and lower limbs.

The second aim was to evaluate the effects of mMeD on the LIPPY subjects’ general health status, the perception of pain, fatigue and the problems that commonly arise during the patients’ daily life.

## 2. Materials and Methods

### 2.1. Study Design and Subjects

In the period between June 2019 and December 2019, we consecutively enrolled in the study all the women who voluntarily came up at the Section of Clinical Nutrition and Nutrigenomics, Department of Biomedicine and Prevention of the University of Rome Tor Vergata for nutritional medical check-up.

According to health status, the patients were separated into two groups. The LIPPY group was represented by women with the diagnosis of lipoedema made at the San Giovanni Battista Hospital in Rome (Italy); the CTRL group included patients not affected by lipoedema. For both groups, inclusion criteria were as follows: Italian Caucasian females older than 18 years old with the body mass index (BMI) > 18.5 kg/m^2^; exclusion criteria were as follows: diagnosis of lymphedema, acute and chronic kidney failure, age over 65, drug use, bariatric surgery and liposuction during the period of treatment, pregnancy and breastfeeding.

According to the inclusion criteria, the subjects eligible for study underwent a medical examination and a complete evaluation of their nutritional status, body composition and basal metabolism at baseline and after four weeks. The mMeD was carried out for four weeks to avoid dropout and ensure maximum adherence to treatment. Moreover, to ensure adherence to the diet, patients were monitored during the four weeks by telephone interview once a week. The patients were asked about their food intake with a 48-h recall, the possible presence of side effects and a general opinion on the satisfaction with the diet.

All the enrolled patients signed a consent form following the principles of the Declaration of Helsinki. The approval by the Ethics Committee of the Calabria Region Center Area Section (Register Protocol No. 146 17/05/2018) was obtained.

### 2.2. Anthropometrics and Body Composition

After a 12-h overnight fast, an anthropometric evaluation was carried out for each patient. Body weight and height were measured using a scale and a stadiometer (Invernizzi, Rome, Italy) while the subject was standing wearing underwear. The data were collected to the nearest 0.1 kg and 0.1 cm, respectively. Neck, waist and hip circumferences were measured with a flexible and non-extensible metric tape.

BMI was calculated as body weight (kg)/height (m)^2^ and classified according to the World Health Organization (WHO) [24]. A waist to hip circumferences ratio (WHR) was evaluated according to the clinical risk thresholds equivalent to WHR  >  0.85 for women [24].

Body composition was evaluated according to the standard method [18]. The patients were asked to remove all clothing (except underwear), shoes and any metal objects. Whole and segmental FM (kg) was evaluated by DXA (Primus, X-ray densitometer; software version 1.2.2, Osteosys Co., Ltd., Guro-gu, Seoul, Korea) [25]. The effective radiation dose for this procedure is about 0.01 mSv. The intra- and inter-subject coefficient of variation (CV% = 100 SD/mean) ranged from 1 to 5%. The coefficients on this instrument for five participants scanned six times over a 9-month period were 2.2% for FM and 1.1% for free fat mass (FFM) and LM.

Total FM percentage (%FM) was calculated as the total body FM (Total FM) divided by the total mass of all tissues including the total body bone (TBBone) as follows:

%FM = (Total FM/(Total FM + Total LM + TBBone)) × 100

According to %FM, subjects are classified as normal weight (NW) lean women with %FM < 30%; pre-obese and obese women with %FM ≥ 30% [25].

Intermuscular adipose tissue (IMAT) was calculated according to Colica et al. with the following formulas: Log (IMAT) = −2.21 + (0.12 × fat) + (−0.0013 × fat2) for women [26].

Bioelectrical impedance analysis (BIA) (BIA101S, Akern/RJL Systems, Florence, Italy) was employed to measure resistance (Rz), reactance (Xc), total body water (TBW, L), extracellular body water (ECW, L) [25].

### 2.3. Indirect Calorimetry

Indirect calorimetry was performed to measure oxygen consumption (VO_2_) and carbon dioxide production (VCO_2_) according to De Lorenzo et al. [27] and to calculate the resting energy expenditure (REE) using a Vyntus CPX Canopy (CareFusion, Höchberg, Germany) with the Sentry Suite™ software (CareFusion, Höchberg, Germany). A gas mixture with 12.0% O_2_, 5.0% CO_2_, balanced with N_2_ was used. After a steady-state condition, when no variation over ± 5% occurred, VO_2_ and VCO_2_ values were recorded.

The measurement was performed after fasting for 12 h. The subjects were asked to lie down on a laboratory bed in a supine position for 25–30 min in a suitable room (quiet, with an ambient temperature of 22 °C).

The REE was determined using the Weir formula [28]:

REE = ((3.94 × VO_2_) + (1.106 × VCO_2_)) × 1.44.

The daily energy requirements were calculated by multiplying REE by the proper physical activity level (PAL) [29].

### 2.4. Dietary Assessments

At baseline and during the mMeD, eating habits of the subjects were evaluated through accurate data collection. Subject’s food intake was assessed with a 3 days/week diet record, 2 weekdays and 1 weekend day, completed for 3 weeks, for a total of 9 days [24].

A food frequency questionnaire was used to identify the weekly frequency of intake of different foods (Appendix A) [30].

### 2.5. Dietary Intervention

All the enrolled subjects received the same hypocaloric mMeD with a caloric restriction of about 20% compared to the daily energy requirements, personalized according to each patient’s energy requirements and LM content. Therefore, the mMeD considered the need of macro- and micronutrient and caloric intake based on the individual characteristics of body composition and basal metabolism. The diet indicated for each day of the week the foods to be consumed divided into 5 meals a day. The average caloric distribution of the meals was as follows: 15%—breakfast, 10%—morning snack, 35%—lunch, 10%—afternoon snack, 30%—dinner.

The mMeD’s daily macronutrient intake was broken down as follows: 40–45% of total kcal/day of carbohydrates, 25–30% of total kcal/day of proteins (>50% of them vegetable-derived), 25–30% of total kcal/day of lipids (in the total daily energy intake: saturated fat < 10%, 6–10% polyunsaturated fatty acids (PUFA), *n*-6/*n*-3 PUFA ratio of 3:1, 15% monounsaturated fatty acids (MUFA), <1% trans-fatty acids) and 25 g of fiber.

The daily protein intake was 2 g/kg of the total LM, according to Colica et al. [26].

The bromatological composition of the dietary intervention was obtained using the diet analyzer software package Dietosystem^®^ (version 12.00.13, DS Medica SRL, Milan, Italy).

The main features of the mMeD are as follows: primarily plant-based foods such as seasonal fruits and vegetables, whole grains, legumes and nuts; replacement of butter with healthy fats such as olive oil; use of herbs and spices instead of salt; foods rich in polyunsaturated fats. Preserved and processed foods such as cold cuts, cured meats and canned products, frozen ready meals, cheese (apart from ricotta), potatoes, high glycemic index (GI) carbohydrates, alcoholic and non-alcoholic sweetened drinks were avoided.

The Mediterranean adequacy index (MAI) [31] was calculated using the ratio of the caloric intake (%kcal/day) derived from carbohydrates and typical Mediterranean foods (like bread, pasta, vegetables, fruit, extra virgin olive oil, fish, red wine) and non-typical ones (like meat, milk and dairy products, eggs, sugar, sweets and alcohol) [32]. MAI values are considered acceptable when the value is >5, and 100% adequate >15. Oxygen radical absorbance capacity (ORAC) of the diet was calculated using the diet analyzer software package Dietosystem^®^ (version 12.00.13, DS Medica SRL, Milan, Italy) to evaluate the protection provided by antioxidant compounds from food [22].

### 2.6. Quality of Life

The quality of life was assessed by using the “European Quality of Life” tool (EQ-5D) [33,34].

The EQ-5D consists of two distinct sections. In the first one, a subjective evaluation for 5 dimensions (mobility, self-care, daily activities, pain/discomfort and anxiety/depression) is requested. For each one, it is possible to choose a level of severity ranging from 1 to 3: level 1 (no problem); level 2 (some trouble); level 3 (extreme limitation). The combination of all the answers forms a 5-digit number that represents the patient’s general health status. The three response levels for each of the 5 dimensions produce a maximum of 243 possible descriptions of the health status and allow highlighting the presence/absence of any problem and its intensity. The second section of the EQ-5D includes a visual analogue scale (VAS) graphically represented by a graduated scale ranging from 0 (the worst possible state of health) to 100 (the best possible state of health), on which a patient indicates her/his perceived level of health. For the present analysis, the scores obtained from the first and the second part at baseline and after four weeks were compared to evaluate any improvement regarding the general state of health as well as the pain, the malaise and the psychological aspects related to anxiety and depression.

### 2.7. Fibromyalgia Assessment Status

Since patients affected by lipoedema usually present pain similar to those of fibromyalgia in their body, the Fibromyalgia Assessment Status (FAS) tool was used to evaluate the effect of the diet on pain and feeling of fatigue [35]. The FAS is a simple self-administered assessment that combines the patient’s evaluation of fatigue and sleep disturbance (2 items, score ranging from 0 to 10) and pain assessed on the basis of 16 non-articular sites listed on the self-assessment pain scale (SAPS) (score ranging from 0 to 3) in a unique tool.

### 2.8. Statistical Analysis

The data collected before statistical evaluations were analyzed for the presence of outliers and for normal distribution with the Shapiro–Wilk test. Outliers were not identified, and the database did not undergo any changes before performing statistical analysis. The data presented are expressed as the means ± standard deviation and as ∆% to evaluate differences between the times. At baseline, the differences between LIPPY and CTRL were assessed by the independent samples t-test and the Mann–Whitney test. Subsequently, the t-Test for related samples or the Wilcoxon rank test were used to assess the presence of differences in the variables examined between the baseline and after four weeks. Conclusively, for each study variable, to compare the trend over time, ∆% was calculated equal to the percentage variation of each parameter calculated as an absolute margin of variation from the baseline value. The differences in ∆% between the baseline and after four weeks among groups were assessed with one-way ANOVA. Results were significant for *p*-value < 0.05. All *p*-values shown are two-tailed. Statistical analysis was performed using IBM SPSS Statistics V25.0 (SPSS, Chicago, IL, USA).

## 3. Results

### 3.1. Dietary Components

At baseline, the results of analysis of dietary components (macro- and micronutrients and nutritional indexes) were calculated using the subject’s food intake assessed with a 3 days/week diet record for three weeks (Table 1). The mean dietary components of the baseline diet and the mMeD (macro- and micronutrients and nutritional indexes) of both groups are shown in Table 1. Significant differences were highlighted for the following parameters: decreased animal proteins (%) (LIPPY: *p* < 0.05, CTRL: *p* < 0.0001), increased total fiber (g) (LIPPY and CTRL: *p* < 0.0001), decreased SFA (g) (LIPPY and CTRL: *p* < 0.05), increased EPA (g) (LIPPY and CTRL: *p* < 0.05), increased DHA (g) (LIPPY: *p* < 0.0001, CTRL: *p* < 0.05), decreased ω6/ω3 ratio (LIPPY and CTLR: *p* < 0.0001), increased K (mg) (LIPPY: *p* < 0.0001, CTRL: *p* < 0.05), increased Fe (mg) (LIPPY: *p* < 0.0001, CTRL: *p* < 0.05), increased Mg (mg) (LIPPY: *p* < 0.0001, CTRL: *p* < 0.05). An increase in the vitamin contents was also observed: vitamin A (µcg) (LIPPY and CTRL: *p* < 0.05), vitamin D (µcg) (LIPPY and CTRL: *p* < 0.05), vitamin C (mg) (LIPPY: *p* < 0.0001, CTRL: *p* < 0.05). Finally, an increased MAI was detected when comparing baseline with the mMeD (LIPPY and CTRL: *p* < 0.0001). No statistical differences were detected between groups.

### 3.2. Anthropometry and Body Composition

Of the 40 enrolled subjects, 34 met the inclusion criteria; five of them declined to participate. Finally, 29 subjects were included in the data analysis and divided into two groups: 14 patients in the LIPPY group and 15 patients in the CTRL group.

**Figure 1:**
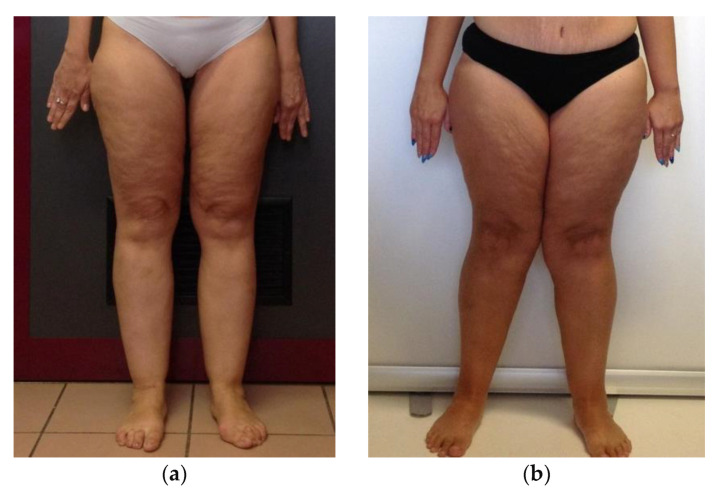

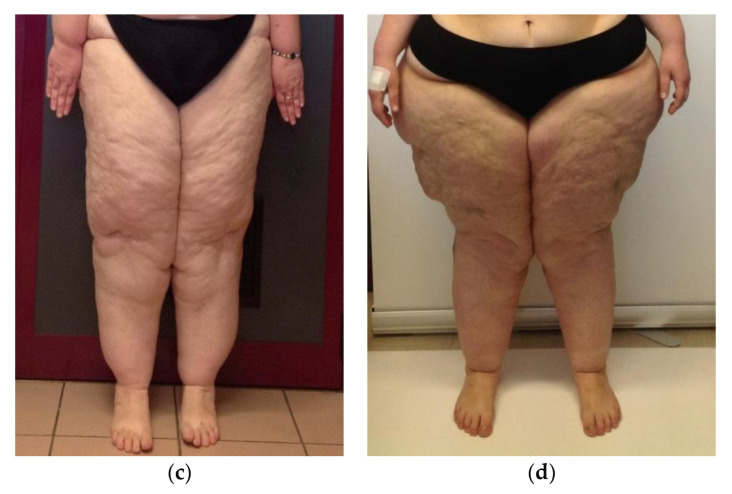
Pictures of different stages of lipoedema. (**a**) Stage 1: the skin is still smooth and appears normal but with pain, bruising and nodules in the fat tissue. (**b**) Stage 2: the skin is characterized by a mattress-like pattern with the presence of fibrosis, development of nodular or mass-like appearance of the subcutaneous fat, lipomas and/or angiolipomas. (**c**); Stage 3 involves the loss in elasticity, inhibition of mobility, inflammation followed by constant and palpable fibrosis. (**d**) Stage 4: presence of both lipoedema and lymphoedema.

Figure 1 depicts different stages of lipoedema identified in the LIPPY group: 14.3% of the patients were in stage 1, 42.8% of them were in stage 2, 28.6% of them were in stage 3, 14.3% of them were in stage 4.

At baseline, no statistical differences were observed between groups for age, height, weight, BMI, circumferences, Rz, TBW parameters. However, WHR, Xc and ECW were significantly different (*p =* 0.029; *p* = 0.001; *p* = 0.012; *p =* 0.006, respectively) between the two groups (data not shown).

The intra- and inter-group (Δ%) comparison of anthropometric, bioimpedance and metabolic parameters at baseline and after four weeks of the mMeD is shown in Table 2. After the mMeD, in the CTRL group, weight, BMI, neck, waist, hip circumference and WHR were significantly reduced (*p* = 0.001; *p* = 0.002; *p* = 0.012; *p* = 0.003; *p* = 0.002; *p* = 0.040). In the LIPPY group, weight and BMI were significantly reduced (*p* = 0.025; *p* = 0.021). For both groups, no other statistical differences were observed after four weeks of the mMeD. A significant difference in the waist circumference Δ% between the LIPPY and CTRL groups was observed (*p* = 0.022).

At baseline, legs’ FM (kg), LM (kg), total mass (kg) and fat region (%) were statistically different between the two groups *(p* = 0.013; *p* = 0.029; *p* = 0.008; *p* = 0.019, respectively).

**Figure 2:**
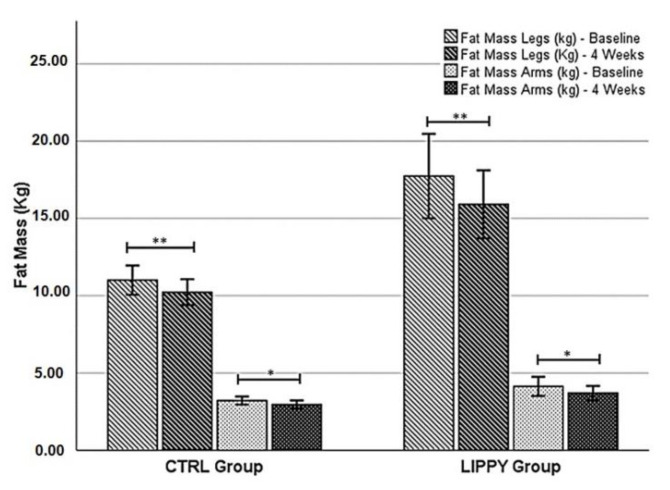
Differences between the baseline and after four weeks of the mMeD in each group for the legs’ and arms’ fat mass. Statistical significance was attributed as * *p* < 0.05; ** *p* < 0.01.

The intra- and inter-group (Δ%) comparison of body composition parameters by DXA at baseline and after four weeks of the mMeD are shown in Table 3. After four weeks of the mMeD, a significant decrease of most of the analyzed parameters was highlighted in the CTRL group. In the LIPPY group, unlike in the CTRL group, the total fat free mass and the legs’ total mass (kg) were significantly reduced (*p* = 0.001; *p* = 0.011). Conversely, for both groups, the arms’ and legs’ FM (kg) had a significant decrease after four weeks of the mMeD (LIPPY: arms, 4.06 ± 1.88 to 3.70 ± 1.47, *p* = 0.048; legs, 18.17 ± 8.52 to 15.91 ± 6.96, *p* = 0.007; CTRL: arms, 3.21 ± 0.99 to 2.96 ± 1.02, *p* = 0.046; legs, 11.01 ± 3.66 to 10.22 ± 3.24, *p* = 0.004) (Figure 2). No difference in Δ% between the groups was found (arms’ FM Δ%: CTRL, −7.79 ± 15.12; LIPPY, −6.66 ± 10.61; *p* = 0.841; legs’ FM Δ%: CTRL, −6.90 ± 6.23; LIPPY, −8.76 ± 7.20; *p* = 0.499).

Comparing Δ% of the parameters between the LIPPY and the CTRL groups at baseline and after the mMeD, significant differences in trunk FM (kg), total body FM (kg), trunk total mass (kg), trunk fat region % and IMAT were observed (*p* = 0.012; *p* = 0.042; *p* = 0.026; *p* = 0.029; *p* = 0.006) (Table 3). 

### 3.3. Quality of Life and Fibromyalgia

The EQ-5D highlighted a significant improvement of the perceived quality of life in the lipoedema patients, with the total score decreasing from an average value of 8.3 ± 1.8 at baseline to a value of 6.9 ± 1.4 after four weeks of treatment (*p* < 0.05). No clinically significant modification were observed regarding the VAS (baseline: 64.7 ± 18.3; 4 weeks: 69.9 ± 18.3; *p* = 0.47), the fibromyalgia severity scale index (*p* = 0.21) and the Fibromyalgia Assessment Status scale (*p* = 0.75). No significant results were observed in the CTRL group related to EQ-5D, VAS, fibromyalgia severity scale index and Fibromyalgia Assessment Status scale, probably because these subjects did not usually experience pain or other discomfort in their daily life (data not shown).

## 4. Discussion

For the improvement of the quality of life of LIPPY patients, it is essential to find a dietary strategy that can be accepted and followed, aimed not only at weight loss and reducing FM in the areas of the lower and upper limbs, but above all at the reduction of pain that is accompanied by the expansion of inflamed subcutaneous tissue and orthostatic edema [36,37]. Moreover, weight gain should be avoided to prevent edema worsening [38].

The main aim of lipoedema treatment should also target factors which negatively influence lipoedema (such as obesity, the presence of lymphatic or venous edema, incorrect insight into the condition and decreased level of physical activity). It was therefore hypothesized that a low-calorie diet plan based on foods rich in antioxidant and anti-inflammatory molecules [22] could contribute to the well-being of LIPPY patients and healthy lifestyle [39]. Therefore, the goal of the mMeD was to reduce consumption of saturated fatty acids and preserved foods [20]. The mMeD included wholesome foods, mostly plant-based, a large number of vegetables and fruits and fermented foods to obtain an increase in ORAC units per day and the right dosage of food antioxidants [22]. Moreover, sugars, chemically modified fats and processed foods were eliminated. Animal proteins were decreased in favor of plant-based proteins (Table 1). The ω6/ω3 ratio was markedly changed with respect to the baseline (LIPPY and CTRL: *p* < 0.0001) thanks to the addition of foods with a high concentration of omega-3 polyunsaturated fatty acids such as fish and nuts that play an important role in the regulatory process of inflammation by promoting an anti-inflammatory effect [21]. As fibers are involved in the modulation process of inflammation, immune system and microbiota equilibrium [22], fiber consumption from fruits and whole grains was significantly increased with respect to the baseline (LIPPY and CTRL: *p* < 0.0001). The mMeD content of molecules with antioxidant and anti-inflammatory activity [32] such as vitamins A (LIPPY and CTRL: *p* < 0.05), C (LIPPY: *p* < 0.0001, CTRL: *p* < 0.05), D (LIPPY and CTRL: *p* < 0.05) and minerals [40,41,42] was significant increased with respect to the baseline, with amelioration of the MAI index (LIPPY and CTRL: *p* < 0.0001).

At baseline, no differences in age and anthropometric parameters were observed between the two groups. However, LIPPY women had significantly higher values of leg compartments with respect to the CTRL group (data not shown), as already expected for this pathology. Moreover, the FM (kg and %) of legs and the FM (%) of arms were higher in the LIPPY group with respect to the CTRL, confirming that lipoedema affects mainly upper and lower limbs. Therefore, an expected result was WHR lower in the LIPPY group than in the CTRL group due to characteristic fat deposition of this disease [43]. Regarding the BIA results, the LIPPY group presented lower Xc values with respect to the CTRL group, and consequently a higher ECW value. This is probably related to increased fat deposition in legs with a consequent expansion of the ECW compartment [44].

In both groups, weight and BMI were significantly decreased after four weeks of the mMeD without differences in Δ% between the two groups (Table 2). In the CTRL group, weight loss was reflected in reduction of all body composition parameters and related circumferences. After the mMeD, LIPPY patients showed only a decrease of FM in upper and lower limbs and a decrease of LM in legs, without significant differences in Δ% with respect to the CTRL group (Table 3). In the CTRL group, we observed that truncal fat was the compartment characterized by the highest loss, as previously demonstrated [25,45]. Moreover, our results do not confirm the statement of Wold et al. [46], according to whom women affected by lipoedema are characterized by low REE [27,47].

Although the loss of FM in the upper and lower limbs was observed in both groups, the statistical non-significance of Δ% reinforces the observed data. For the first time a diet such as the mMeD has been shown to be effective in reducing FM in the typical points of lipoedema, such as in women not affected by this disease. Loss of upper and lower limbs’ fat can probably be the key to decreasing symptoms in lipoedema. For the first time, an increase in the LIPPY patients’ ability to perform various daily physical activities after a diet therapy was observed. According to the EQ-5D scale results, the possibility of those patients to perform simple daily activities with less fatigue, pain and anxiety is highlighted due to their body condition probably improving their perception of the quality of life. This might reflect a positive response to the mMeD, allowing a higher quality of daily life [11].

The limitations of the study were the small number of participants, though the number of patients is sufficient for rare pathology studies such as lipoedema-and short intervention period. Therefore, we enrolled all the available patients without differentiating them by disease stages and determining as a consequence a wide variance in BMI. Since lipoedema is a rare adipose disorder characterized by expansion of the SAT, the BMI cannot be considered an indicator of obesity, as obesity itself may or may not overlap. The intervention period was a mandatory choice because it was the period of greatest adherence to the prescribed diet therapy. Despite the bias linked to the self-reporting of dietary intake, the data discussed make it possible to notice that the mMeD showed increased antioxidant and anti-inflammatory nutritional indexes. However, more data are needed for a larger population, and long-term interventions are required. Moreover, future studies of different types of diet therapies such as low-carb, high-fat or focused on specific antioxidant micronutrients, as compared to a control group following a normal diet, will be necessary to evaluate efficacy of pain management as related to weight and FM loss. Finally, in order to evaluate the antioxidant and anti-inflammatory effect of the diet, further studies directly measuring the inflammatory and oxidative status of the patients should be performed.

In conclusion, the strength of this study was the adherence of LIPPY patients to the mMeD; this improved overall nutritional status and quality of life, reducing weight, arms’ and legs’ FM. Although more studies are recommended to investigate whether the increase in the antioxidant capacity corresponds to weight loss, our study highlighted that the mMeD with high values of MAI [48] and ORAC [22] could be a nutritional strategy for lipoedema treatment.

## Figures and Tables

**Table 1:** Dietary components (macro- and micronutrients) and nutritional indexes of the mMeD with respect to the baseline diet.

	LIPPY		CTRL	
	Baseline	mMeD	Baseline	mMeD
	Mean ± SD	Mean ± SD	Mean ± SD	Mean ± SD
Energy (Kcal)	1536.0 ± 320.8	1370.4 ± 222.8 *	1570.7 ± 301.7	1494.1 ± 171.5
Proteins (% Kcal)	17.6 ± 3.8	27.2 ± 4.4 **	20 ± 3.7	22.5 ± 2.04
Vegetable proteins (% Kcal)	28.2 ± 9.4	30.2 ± 4.6	28.5 ± 8	32 ± 8
Animal proteins (% Kcal)	71.8 ± 9.4	62 ± 5.3 *	67.4 ± 8.9	52.6 ± 5.2 **
Carbohydrates (% Kcal)	37.5 ± 11.8	40.2 ± 1.6	43.9 ± 7.1	43.8 ± 3
Sugars (% Kcal)	13.6 ± 4	14.6 ± 1.17	15.9 ± 4.6	16.3 ± 2
Total fiber (g)	13.8 ± 6	26.4 ± 4 **	19.7 ± 4.3	28.9 ± 3.6 **
Lipids (% Kcal)	44.4 ± 9.6	31.9 ± 3.6 *	36.1 ± 7	33.5 ± 3.1
SFA (g)	18.2 ± 6.9	9.4 ± 2.2 *	17.5 ± 7.9	12.6 ± 3 *
PUFA (g)	8.6 ± 3.4	9.05 ± 4	6.9 ± 1.7	8.2 ± 1.5 *
PUFA/SFA	0.47 ± 0.2	0.93 ± 0.2 **	1.7 ± 3	0.64 ± 0.2
MUFA (g)	36.1 ± 13.4	23.4 ± 5.9 *	32 ± 9.7	28.1 ± 4.9
EPA (g)	0.13 ± 0.21	0.53 ± 0.27 *	0.10 ± 0.20	0.40 ± 0.11 *
DHA (g)	0.20 ± 0.40	1.1 ± 0.46 **	0.22 ± 0.54	0.62 ± 0.24 *
ω6/ω3	8.04 ± 3.2	2.6 ± 0.54 **	6.1 ± 1.8	3.5 ± 1 **
Oleic acid (g)	29.7 ± 12	23.3 ± 5.5	30.7 ± 9.3	27 ± 4.7
Na (mg)	1210 ± 996.7	957.4 ± 236.1	1435.5 ± 677.4	1278.8 ± 203.7
K (mg)	2270.7 ± 655.5	3549.1 ± 342.1 **	2871.8 ± 631.1	3637.1 ± 390.2 *
Fe (mg)	9.7 ± 3.2	15.1 ± 2.17 **	10.5 ± 2	13.6 ± 1.81 *
Mg (mg)	162.7 ± 75.5	295.8 ± 37.2 **	227.5 ± 94	304.1 ± 48.3 *
Vit. A (µcg)	764.3 ± 329.7	1091.3 ± 71.2 *	930.6 ± 306	1281.2 ± 455.9 *
Vit. C (mg)	112 ± 48.5	190.2 ± 14.7 **	145.4 ± 62.20	224.5 ± 38.2 *
Vit. D (µcg)	4.3 ± 4.4	7.63 ± 2.5 *	2.3 ± 3.8	5.3 ± 2 *
Vit. E (mg)	11.7 ± 2.2	12.6 ± 2	5.3 ± 11.8	14.7 ± 1.6
ORAC (µmol)	2423.0 ± 1814.0	13538.0 ± 1517.0 **	11759.0 ± 6910.0	14105.0 ± 1584.0
MAI	1.4 ± 0.7	14.7 ± 0.7 **	1.45 ± 1.07	14.06 ± 1.9 **

SFA, saturated fatty acid; PUFA, polyunsaturated fatty acid; MUFA, monounsaturated fatty acid; EPA, eicosapentaenoic acid; DHA, docosahexaenoic acid; Na, sodium; K, potassium; Fe, iron; Mg, magnesium; Vit., vitamin; ORAC, oxygen radical absorbance capacity; MAI, Mediterranean adequacy index; mMeD, modified Mediterranean diet therapy. The paired t-test was performed to compare dietary intake of the baseline diet and of the mMeD. * *p* < 0.05; ** p < 0.0001.

**Table 2:** Comparison of anthropometric, indirect calorimetry and bioimpedance variables between the baseline and after four weeks.

Parameters	CTRL			LIPPY			Δ% Baseline–4 Weeks		
	Baseline	4 Weeks		Baseline	4 Weeks		CTRL	LIPPY	
	Mean ± SD	Mean ± SD	*p*	Mean ± SD	Mean ± SD	*p*	Mean ± SD	Mean ± SD	*p*
Weight (kg)	73.45 ± 14.50	70.21 ± 14.09	0.001 **†	91.06 ± 28.63	88.1 ± 27.7	0.025 *†	−4.37 ± 3.94	−3.04 ± 4.75	0.418
BMI (kg/m^2^)	27.52 ± 5.22	26.38 ± 4.99	0.002 **†	35.50 ± 12.17	34.36 ± 11.84	0.021 *†	−4.05 ± 4.03	−3.04 ± 4.75	0.540
Neck C. (cm)	35.50 ± 3.16	34.77 ± 3.08	0.012 *	37.21 ± 3.59	36.79 ± 2.96	0.443	−2.12 ± 2.45	−0.89 ± 5.32	0.469
Waist C. (cm)	85.12 ± 13.37	80.24 ± 10.67	0.003 **	87.18 ± 16.08	85.44 ± 14.44	0.115	−5.43 ± 4.33	−1.66 ± 3.84	0.022 *
Hip C. (cm)	108.08 ± 9.87	105.19 ± 9.06	0.002 **†	122.11 ± 23.31	120.68 ± 21.76	0.250†	−2.59 ± 2.55	−0.95 ± 3.89	0.198
WHR	0.78 ± 0.08	0.76 ± 0.07	0.040 *	0.72 ± 0.07	0.71 ± 0.06	0.612	−3.00 ± 4.36	−0.60 ± 5.15	0.194
VO2 (ml/min)	218.87 ± 31.61	213.57 ± 36.55	0.323	217.14 ± 46.08	213.17 ± 40.93	0.515	−2.02 ± 7.01	−1.57 ± 14.29	0.919
VCO2 (ml/min)	182.13 ± 29.55	181.36 ± 31.38	0.752	173.57 ± 41.52	172.75 ± 32.64	0.972	0.13 ± 2.51	2.36 ± 20.9	0.694
MREE (kcal)	1508.00 ± 247.11	1502.79 ± 260.49	0.677	1479.21 ± 321.51	1454.83 ± 281.4	0.633	0.24 ± 2.68	−0.88 ± 15.18	0.789
Rz	561.60 ± 75.80	569.33 ± 77.56	0.320	507.64 ± 86.63	510 ± 94.72	0.779	1.53 ± 5.02	0.43 ± 6.25	0.603
Xc	59.87 ± 9.62	58.27 ± 9.60	0.411	46.64 ± 9.37	49.71 ± 14.62	0.344	−2.21 ± 11.29	7.07 ± 24.27	0.193
TBW (L)	34.89 ± 4.66	34.37 ± 4.53	0.800†	37.99 ± 5.84	37.79 ± 5.97	0.396 †	−1.43 ± 2.82	−0.54 ± 3.7	0.469
ECW (L)	15.80 ± 2.08	16.00 ± 2.22	0.522	18.88 ± 3.37	18.35 ± 3.73	0.433	1.48 ± 8.03	−2.49 ± 13.00	0.328
BCM (kg)	25.93 ± 4.98	24.82 ± 3.65	0.110	25.12 ± 3.66	25.68 ± 4.85	0.559	−3.45 ± 7.77	2.39 ± 14.53	0.184
PA (°)	6.13 ± 1.08	5.85 ± 0.61	0.008 **†	5.24 ± 0.59	5.54 ± 1.24	0.683 †	−3.01 ± 11.44	6.15 ± 22.28	0.171
BCMI (kg/m^2^)	9.73 ± 1.78	9.31 ± 1.24	0.153 †	9.76 ± 1.71	10.08 ± 2.31	0.875 †	−3.48 ± 7.62	3.29 ± 16.70	0.166

Differences between the baseline and after four weeks after the mMeD in each group. Parameters are presented as the means ± standard deviation and Δ% between the baseline and after four weeks. Differences between the baseline and after four weeks in each group were compared by the related samples t-test and (†) the Wilcoxon test, while Δ% baseline–4 weeks differences between the groups were compared by one-way ANOVA. Statistical significance was attributed as * *p* < 0.05; ** *p* < 0.01. BCM, body cell mass; BCMI, body cell mass index; BMI, body mass index; C., circumference; CTRL, control group; ECW, extracellular water; LIPPY, lipoedema group; MREE, measured resting energy expenditure; PA, phase angle; Rz, resistance; SD, standard deviation; TBW, total body water; VCO2, volumes of carbon dioxide; VO2, volumes of oxygen; WHR: waist-to-hip Ratio; Xc, reactance.

**Table 3:** Comparison of body composition variables between the baseline and after four weeks.

Parameters	CTRL			LIPPY			Δ% Baseline−4 Weeks		
	Baseline	4 Weeks		Baseline	4 Weeks		CTRL	LIPPY	
	Mean ± SD	Mean ± SD	*p*	Mean ± SD	Mean ± SD	*p*	Mean ± SD	Mean ± SD	*p*
Fat mass, trunk (kg)	14.09 ± 5.97	12.39 ± 6.03	0.001 **	18.66 ± 11.71	17.64 ± 11.23	0.220	−13.77 ± 12.44	−0.81 ± 10.17	0.012 *
Fat mass, android (kg)	2.26 ± 1.14	2.05 ± 1.14	0.003 **	3.08 ± 2.06	3.08 ± 2.47	0.717	−11.95 ± 13.31	−1.14 ± 17.68	0.094
Fat mass, gynoid (kg)	5.43 ± 1.74	4.92 ± 1.67	0.001 **†	7.81 ± 4.33	7.16 ± 3.68	0.074†	−9.88 ± 7.44	−4.27 ± 8.49	0.094
Fat mass, total body (kg)	29.2 ± 9.8	26.4 ± 9.49	0.001 **†	42.02 ± 21.94	39.25 ± 20.24	0.114†	−9.94 ± 8.86	−3.10 ± 5.65	0.042 *
Lean mass, arms (kg)	4.49 ± 0.97	4.4 0± 1.02	0.117 †	4.41 ± 0.78	4.06 ± 0.68	0.225†	−2.16 ± 4.87	−4.52 ± 11.13	0.473
Lean mass, legs (kg)	14.82 ± 2.58	14.46 ± 2.64	0.023 *	17.13 ± 2.83	15.87 ± 2.81	0.083	−2.49 ± 3.81	−4.45 ± 6.96	0.373
Lean mass, trunk (kg)	19.31 ± 2.88	19.47 ± 2.95	0.516	21.42 ± 4.22	21.42 ± 4.26	0.126	0.90 ± 5.00	3.47 ± 6.13	0.263
Lean mass, total body (kg)	41.74 ± 6.27	41.38 ± 6.32	0.367	46.65 ± 7.99	45.74 ± 7.43	0.366	−0.85 ± 3.52	1.89 ± 4.43	0.099
Total mass, arms (kg)	8.01 ± 1.82	7.65 ± 1.89	0.043 *	8.74 ± 2.54	8.02 ± 1.93	0.083	−4.31 ± 7.90	−5.94 ± 8.70	0.632
Total mass, legs (kg)	26.65 ± 5.58	26.18 ± 5.06	0.485	36.15 ± 10.62	32.64 ± 9.53	0.011 *	−1.01 ± 11.38	−6.87 ± 5.54	0.145
Total mass, trunk (kg)	34.13 ± 7.73	32.58 ± 7.66	0.006 **	40.86 ± 15.33	39.93 ± 14.93	0.917	−4.55 ± 5.44	1.12 ± 6.40	0.026 *
Total mass, android (kg)	5.12 ± 1.45	4.93 ± 1.45	0.028 *	6.38 ± 2.63	6.53 ± 3.69	0.456	−4.00 ± 6.14	3.29 ± 17.09	0.141
Total mass, gynoid (kg)	12.12 ± 2.4	11.49 ± 2.36	0.001 **	15.27 ± 5.58	14.27 ± 4.78	0.293	−5.17 ± 4.81	−2.85 ± 8.14	0.380
Fat region %, arms	39.75 ± 5.61	38.06 ± 6.79	0.106	44.16 ± 10.39	44.61 ± 9.55	0.467	−4.27 ± 9.87	−0.72 ± 6.35	0.326
Fat region %, legs	40.6 ± 6.32	39.44 ± 6.20	0.029 *	48.19 ± 9.48	47.3 ± 8.43	0.183	−2.86 ± 4.67	−1.57 ± 4.36	0.492
Fat region %, trunk	39.92 ± 9.40	36.41 ± 10.6	0.001 **	41.61 ± 13.91	40.78 ± 12.74	0.123	−9.92 ± 9.84	−2.03 ± 4.90	0.029 *
Fat region %, android	42.08 ± 11.31	39.22 ± 12.72	0.001 **	43.04 ± 16.63	41.8 ± 14.67	0.071	−8.6 ± 10.37	−4.21 ± 8.18	0.273
Fat region %, gynoid	43.95 ± 6.90	41.88 ± 7.39	0.003 **	48.20 ± 11.03	47.65 ± 9.34	0.121	−4.98 ± 5.93	−1.84 ± 4.29	0.164
Fat region %, total body	39.02 ± 7.13	36.78 ± 7.57	0.002 **	42.78 ± 11.63	42.5 ± 10.08	0.796	−6.07 ± 6.45	1.15 ± 11.92	0.061
IMAT	1.14 ± 0.43	1.02 ± 0.45	0.004 **	1.37 ± 0.54	1.35 ± 0.54	0.674	−11.57 ± 11.88	0.74 ± 5.38	0.006 **

Differences between the baseline and after four weeks of the mMeD in each group. Parameters are presented as the means ± standard deviation and Δ% between the baseline and after four weeks. Differences between the baseline and after four weeks in each group were compared by the related samples t-test and (†) the Wilcoxon test, while Δ% Baseline–4 weeks differences between the groups were compared by one-way ANOVA. Statistical significance was attributed as * *p* < 0.05; ** *p* < 0.01. CTRL, control group; IMAT, intermuscular adipose tissue; LIPPY, lipoedema group; SD, standard deviation.

## Data Availability

The data presented in this study are available on request from the corresponding author. The data are not publicly available due to privacy.

## Abbreviations

BCM	Body Cell Mass
BCMI	Body Cell Mass Index
BIA	Bioelectrical impedance analysis
BMI	Body Mass Index
CTRL	Control Group
DHA	Docosahexaenoic Acid
DXA	Dual-Energy X-Ray Absorptiometry
ECW	Extracellular Water
EPA	Eicosapentaenoic Acid
Fe	Iron
FFM	Free Fat Mass
FM	Fat Mass
IMAT	Intermuscular Adipose Tissue
K	Potassium
LIPPY	Lipoedema Group
LM	Lean Mass
MAI	Mediterranean Adequacy Index
mMeD	Modified Mediterranean Diet Therapy
Mg	Magnesium
MREE	Measured Resting Energy Expenditure
MUFA	Monounsaturated Fatty Acid
Na	Sodium
ORAC	Oxygen Radical Absorbance Capacity
PA	Phase Angle
PUFA	Polyunsaturated Fatty Acid
Rz	Resistance
Xc	Reactance
SFA	Saturated Fatty Acid
TBW	Total Body Water
VCO2	Volumes of Carbon Dioxide
Vit.	Vitamin
VO2	Volumes of Oxygen
WHR	Waist-to-Hip Ratio

## Appendix A. Medium-Length FFQs

### MEDIUM-LENGTH FOOD FREQUENCY QUESTIONNAIRE

**A. DRINKS**						
1. Do you drink **COFFEE**? Yes ☐ No, never. ☐						
	*How many times per day?* ………					
2. Do you drink **ALCOHOL DRINKS**? Yes ☐ No, never. ☐						
	*How much?*	*Wine:*	*0.15 L* ☐	*0.20 L* ☐	*0.25 L* ☐	
		*Beer:*	*0.23 L* ☐	*0.33 L* ☐	*0.45 L* ☐	*0.66 L* ☐
		*Strong alcohol:*	*0.03 L* ☐	*0.13 L* ☐	*0.20 L* ☐	*0.30 L* ☐
	*How many times per week?* ……… *Less than once a week*. ☐					
3. Do you drink **SOFT DRINKS** (coke, soda…) ? Yes ☐ No, never. ☐						
	*How many times per day?* ………					
**B. MILK and DAIRY PRODUCTS**						
4. Do you drink **MILK**? Yes ☐ No, never. ☐						
	*Which kind?*	*Whole* ☐	*Semi-skimmed* ☐	*Skimmed* ☐		
	*How much?*	*Small (120 mL)* ☐	*Medium (200 mL)* ☐	*Large (300 mL)* ☐		
	*How many times per week?* ……… *Less than once a week*. ☐					
5. Do you eat **Yogurt**? Yes ☐ No, never. ☐						
	*How much?*	*Small (125 g)* ☐	*Medium (150 g)* ☐	*Large (180 g)* ☐		
	*How many times per week?* ……… *Less than once a week*. ☐					
6. Do you eat **CHEESE**? Yes ☐ No, never. ☐						
	*How many times per week?* ……… *Less than once a week*.					
	**6.1. HARD CHEESE***(e.g., parmesan, sheep’s milk, Swiss)* ? Yes ☐ No, never. ☐					
	*How much?*	*Small serving (30 g)* ☐	*Medium serving (50 g)* ☐	*Large serving (70 g)* ☐		
	*How many times per week?* ……… *Less than once a week*. ☐					
	**6.2. SOFT CHEESE**? Yes ☐ No, never. ☐					
	*How much?*	*Small serving (50 g)* ☐	*Medium serving (70 g)* ☐	*Large serving (100 g)* ☐		
	*How many times per week?* ……… *Less than once a week*. ☐					
	**6.3. MOZZARELLA**? Yes ☐ No, never. ☐					
	*How much?*	*Small serving (60 g)* ☐	*Medium serving (125 g)* ☐	*Large serving (200 g)* ☐		
	*How many times per week?* ……… *Less than once a week*. ☐					
	**6.4. COTTAGE CHEESE?** Yes ☐ No, never. ☐					
	*How much?*	*Small serving (100 g)* ☐	*Medium serving (150 g)* ☐	*Large serving (200 g)* ☐		
	*How many times per week?* ……… *Less than once a week*. ☐					
**C. MEAT, FISH and EGGS**						
7. Do you eat **RED MEAT**? Yes ☐ No, never. ☐						
	*How much?*	*Small serving (80 g)* ☐	*Medium serving (120 g)* ☐	*Large serving (200 g)* ☐		
	*How many times per week?* ……… *Less than once a week*. ☐					
8. Do you eat **POULTRY**? Yes ☐ No, never. ☐						
	*How much?*	*Small serving (80 g)* ☐	*Medium serving (120 g)* ☐	*Large serving (160 g)* ☐		
	*How many times per week?* ……… *Less than once a week*. ☐					
9. Do you eat **PORK**? Yes ☐ No, never. ☐						
	*How much?*	*Small serving (50 g)* ☐	*Medium serving (100 g)* ☐	*Large serving (150 g)* ☐		
	*How many times per week?* ……… *Less than once a week*. ☐					
10. Do you eat **FISH**? Yes ☐ No, never. ☐						
	*How much?*	*Small serving (100 g)* ☐	*Medium serving (150 g)* ☐	*Large serving (200 g)* ☐		
	*How many times per week?* ……… *Less than once a week*. ☐					
11. Do you eat **CURED MEATS AND SALAMI**? Yes ☐ No, never. ☐						
	*How much?*					
	***CURED MEAT:***	*Small serving (20 g)* ☐	*Medium serving (40 g)* ☐	*Large serving (80 g)* ☐		
	***SALAMI:***	*Small serving (15 g)* ☐	*Medium serving (30 g)* ☐	*Large serving (50 g)* ☐		
	*How many times per week?* ……… *Less than once a week*. ☐					
12. Do you eat **EGGS**? Yes ☐ No, never. ☐						
	*How many times per week?* ……… *Less than once a week*. ☐					
**D. CEREALS**						
13. Do you eat **PASTA** or **RICE**? Yes ☐ No, never. ☐						
	*How much?*	*Small serving (40–50 g)* ☐	*Medium serving (60–80 g)* ☐	*Large serving (100–120 g)* ☐		
	*How many times per week?* ……… *Less than once a week*. ☐					
14. Do you eat **BREAD**? Yes ☐ No, never. ☐						
	*How much?*	*Small serving (30 g)* ☐	*Medium serving (50 g)* ☐	*Large serving (100 g)* ☐		
	*How many times per week?* ……… *Less than once a week*. ☐					
15. Do you eat **BAKERY PRODUCTS**? Yes ☐ No, never. ☐						
	*How much?*	*Small serving (15 g)* ☐	*Medium serving (30 g)* ☐	*Large serving (45 g)* ☐		
	*How many times per week?* ……… *Less than once a week*. ☐					
16. Do you eat **POTATOES**? Yes ☐ No, never. ☐						
	*How much?*	*Small serving (100 g)* ☐	*Medium serving (150 g)* ☐	*Large serving (200 g)* ☐		
	*How many times per week?* ……… *Less than once a week*. ☐					
17. Do you eat **PIZZA**? Yes ☐ No, never. ☐						
	*How much?*	*Small serving (100 g)* ☐	*Medium serving (150 g)* ☐	*Large serving (300 g)* ☐		
	*How many times per week?* ……… *Less than once a week*. ☐					
**E. VEGETABLES, LEGUMES and FRUIT**						
18. Do you eat **VEGETABLES** or **GREENS**? Yes ☐ No, never. ☐						
	*How much?*	*Small serving (100 g)* ☐	*Medium serving (150 g)* ☐	*Large serving (300 g)* ☐		
	*How many times per week?* ……… *Less than once a week*. ☐					
19. Do you eat **LEGUMES** *(beans, peas)* ? Yes ☐ No, never. ☐						
	*How much?*					
	***DRIED:***	*Small serving (30 g)* ☐	*Medium serving (50 g)* ☐	*Large serving (70 g)* ☐		
	***FRESH:***	*Small serving (75 g)* ☐	*Medium serving (125g)* ☐	*Large serving (175 g)* ☐		
	*How many times per week?* ……… *Less than once a week*. ☐					
20. Do you eat **FRESH FRUIT**? Yes ☐ No, never. ☐						
	*How much?*	*Small serving (100 g)* ☐	*Medium serving (150 g)* ☐	*Large serving (200 g)* ☐		
	*How many times per week?* ……… *Less than once a week*. ☐					
**F. FATTY DRESSINGS**						
21. Do you eat **OLIVE OIL**? Yes ☐ No, never. ☐						
	*How much?*	*1 teaspoon (5 g)* ☐	*2 teaspoons (10 g)* ☐	*2 teaspoons (15 g)* ☐		
	*How many times per week?* ……… *Less than once a week*. ☐					
22. Do you eat **SEED OIL**? Yes ☐ No, never. ☐						
	*How much?*	*1 teaspoon (5 g)* ☐	*2 teaspoons (10 g)* ☐	*2 teaspoons (15 g)* ☐		
	*How many times per week?* ……… *Less than once a week*. ☐					
23. Do you eat **BUTTER**? Yes ☐ No, never. ☐						
	*How much?*	*Small serving (5 g)* ☐	*Medium serving (10 g)* ☐	*Large serving (15 g)* ☐		
	*How many times per week?* ……… *Less than once a week*. ☐					
24. Do you eat **MARGARINE**? Yes ☐ No, never. ☐						
	*How much?*	*Small serving (5 g)* ☐	*Medium serving (10 g)* ☐	*Large serving (15 g)* ☐		
	*How many times per week?* ……… *Less than once a week*. ☐					
**G. OTHER**						
25. Do you eat **SWEETS**? Yes ☐ No, never. ☐						
	*How much?*	*Small serving (30 g)* ☐	*Medium serving (50 g)* ☐	*Large serving (70 g)* ☐		
	*How many times per week?* ……… *Less than once a week*. ☐					
26. Do you eat **FRIED FOODS**? Yes ☐ No, never. ☐						
	*How many times per week?* ……… *Less than once a week*. ☐					
27. Do you eat **FAST FOOD**? Yes ☐ No, never. ☐						
	*Which kind?*					
	***HAMBERGER:***	*Medium serving (120 g)* ☐	*Large serving (210 g)* ☐			
	***Hot Dog:***	*Medium serving (120 g)* ☐	*Large serving (210 g)* ☐			
	***LOCAL STREET FOOD:***					
	*Panelle sandwich*	*Medium serving (200 g)* ☐				
	*Spleen sandwich*	*Medium serving (250 g)* ☐				
	*How many times per week?* ……… *Less than once a week*. ☐					

## Appendix B

**Table A1:** Procedure used to calculate energy and nutrients content from FFQ.

Categories	Items	Scotti-Bassani Atlas Reference (Table Number)	MetaDieta Software Selection
Drinks	Coffee	Cup (n.99)	Espresso
	Wine	Glasses 2 (n.94)	Red wine
	Beer	Bottles and can 1 (n.95)	Lager beer
	Strong drinks	Glasses 1 (n.93)	Vodka
	Soft drinks	Bottles and can 2 (n.95)	Coke
Milk and dairy products	Milk	Milk (n.33)	Semi-skimmed milk
	Yogurt	Yogurt (n.35)	Whole milk yogurt
	Hard cheese	Cheese 2 (n.31)	Standard hard cheese serving
	Soft cheese	Cheese 1 (n.30)	Standard soft cheese serving
	Mozzarella	Mozzarella (n.34)	Mozzarella
	Cottage cheese	Cottage cheese (n.32)	Cottage cheese
Meat, fish and eggs	Red meat	Beef steak (n.40)	Semi-fat beef
	Poultry	Chicken breast (n.42)	Chicken breast
	Pork	Pork steak (n.40)	Pork Sirloin Chop
	Fish	Cod fillet (n.54)	Cod fillet
	Cured meat	Ham (n.46)	Ham
	Salami	Salami (n.48)	Salami
	Eggs	Omelette (n.36)	Chicken eggs
Cereals	Pasta/rice	Pasta and tomato sauce (n.22)	Semolina pasta
	Bread	Bread (n.4)	Bread Bread (n.4) Bread
	Bakery products	Crackers (n.1)Bread sticks (n.3)	Crackers, bread sticks
	Potatoes	Boiled potatoes (n.28)	Boiled potatoes
	Pizza	Pizza (n. 8)	Pizza tomato and mozzarella
Vegetables, legumes and fruits	Vegetables/greenS	Vegetable and greens (n. 61–71)	Lettuce, spinach, eggplant, zucchini, carrots
	Dried legumes	Beans (n.26)	Beans
	Fresh legumes	Peas (n.29)	Peas
	Fresh Fruits	Fresh fruit (n.72–80)	Seasonal fresh fruit
Fatty dressings	Olive oil	Spoons, spoons and ladles (n.98)	Extra-virgin olive oil
	Seed oil	Spoons, spoons and ladles (n.98)	Sunflower seed oil
	Butter	Fat dressings (n.92)	Butter
	Margarine	Fat dressings (n.92)	Margarine
Other	Sweets	Sweets (n.87, 88)	Standard sweets serving
	Fried foods	French fries (n.27)	French fries
	Fast food		
	Hamburger	Hamburger sandwich (n.82)	Hamburger sandwich
	Hot dog		Hot dog
	Local street food		Panelle sandwich and spleen sandwich

Each item was recognized by the interviewee in the food atlas, and then a pre-specified corresponding referral food was considered for calculations in the MetaDieta software.
